# Domestic cats and dogs create a landscape of fear for pest rodents around rural homesteads

**DOI:** 10.1371/journal.pone.0171593

**Published:** 2017-02-03

**Authors:** Themb’alilahlwa A. M. Mahlaba, Ara Monadjem, Robert McCleery, Steven R. Belmain

**Affiliations:** 1 Department of Biological Sciences, University of Swaziland, Private Bag 4, Kwaluseni, Swaziland; 2 Mammal Research Institute, Department of Zoology & Entomology, University of Pretoria, Private Bag 20, Hatfield, Pretoria, South Africa; 3 Department of Wildlife Ecology and Conservation, University of Florida, Gainesville, FL, United States of America; 4 Natural Resources Institute, University of Greenwich, Chatham Maritime, Kent, United Kingdom; University of Southern Queensland, AUSTRALIA

## Abstract

Using domestic predators such as cats to control rodent pest problems around farms and homesteads is common across the world. However, practical scientific evidence on the impact of such biological control in agricultural settings is often lacking. We tested whether the presence of domestic cats and/or dogs in rural homesteads would affect the foraging behaviour of pest rodents. We estimated giving up densities (GUDs) from established feeding patches and estimated relative rodent activity using tracking tiles at 40 homesteads across four agricultural communities. We found that the presence of cats and dogs at the same homestead significantly reduced activity and increased GUDs (i.e. increased perception of foraging cost) of pest rodent species. However, if only cats or dogs alone were present at the homestead there was no observed difference in rodent foraging activity in comparison to homesteads with no cats or dogs. Our results suggest that pest rodent activity can be discouraged through the presence of domestic predators. When different types of predator are present together they likely create a heightened landscape of fear for foraging rodents.

## Introduction

In the evolutionary arms race between predators and their prey, many animals have developed innate and learned behaviours to avoid predation [1]. The impact of predators on the behaviour and physiology of their prey has been the topic of several reviews which highlight opportunities for exploiting these dynamics for pest management [2,3,4]. Although much laboratory research has been able to demonstrate quite clear effects of predatory odours from urine, faeces, fur, skin and anal glands on the behaviour and physiology of prey animals [5,6,7], understanding these dynamics under field conditions has been challenging due to often complex behaviours, habitats, intra-specific competition and habituation [2,3,8]. Despite the once promising laboratory results, the use of extracted or synthesised predator odours [9] have not become widely used for the management of wildlife likely because of habituation to them.

Optimising the amount of time animals spend foraging is an important life strategy directly related to an animal’s level of fitness; the risk of predation when foraging is one element that must be considered. By monitoring the time spent foraging it is possible to elucidate the practical effects of predators on a pest’s perception of risk [10]. The optimisation of foraging behaviour of animals was developed as the Marginal Value Theorem (MVT) [11]. The MVT postulates that a foraging animal assumes that food items occur in clumps and that its food intake decreases along with the time spent in that exact patch. Foragers balance the benefit of energetic reward and the cost of predation when making foraging decisions [12]. Based on those cost-benefit assessments on forthcoming yield of the current patch versus the future yield that could be obtained by moving on to another patch, the forager predicts the value of the patch and makes decisions on whether to depart to the next food patch [13]. By creating food patches and assessing the amount of food left after foraging, the giving-up density (GUD) of a food source becomes a measurable unit [14]. The GUD resembles the perceived costs of foraging on that location. The more food left in a patch after the departure of an animal, the higher the GUD, indicating high costs [15]. With respect to foraging costs, every so often prey need to sacrifice food for safety, and this has been termed the “landscape of fear” [16]. Rodents can assess predation risk during foraging behaviour by indirect cues (e.g. foraging habitat, weather, light levels) or direct cues (predator urine) [17]. Using GUDs in this way has received much recent attention in terms of understanding predator-prey dynamics [18,19].

Cats (*Felis catus*) have been associated with humans for thousands of years, having been domesticated in the Near East around 9,500 years ago [20,21]. It is speculated that wild cats were drawn to human settlements by the abundance of pest rodents associated with the farming and storing of grain. Dogs (*Canis familiaris*) were domesticated even earlier and may have originally been drawn to pre-agricultural hunter-gatherer societies by the build-up of refuse around these camps [22]. Members of the cat genus *Felis* are typically solitary, ambush predators that feed on a wide variety of small vertebrate prey [22]. Feral cats in urban centres typically forage alone and include rats and mice in their diet [23]. In contrast, feral dogs are descended from the social wolf and may hunt in packs, running down prey over long distances [22]. Feral dogs may include small mammals in their diet [24], but more typically subsist on refuse [25]. Hence, domesticated cats and dogs, although both able to feed on pest rodents [24], differ in their hunting techniques and therefore exert different selection pressures on rodent pest populations.

Although there is much anecdotal support from farmer surveys [26,27,28] for the role of domestic cats to control rodent pests, previous research investigating the role of dogs and cats on rodent control is more equivocal [23,24,29,30,31]. However, applying the analytical power of GUDs to help understand the role of domestic predators on domestic rodent pests could help develop innovative strategies whereby pest management attempts to manipulate the landscape of fear to the detriment of rodent fitness. Thus the aims of the current study are to evaluate the anecdotal claims made by rural farming homesteads that cats and dogs have an impact on rodent pests [32]. We aim to do this by assessing relative rodent activity and foraging risk across a replicated comparative trial. This is the first time that the GUD has been applied to a domestic rodent management context. We predict that the presence of cats (alone or with dogs) will reduce pest rodent activity and increase their GUDs compared with their absence. Furthermore, we predict that the presence of dogs alone will have similar effects compared with the absence of cats and dogs.

## Methods

### Study site

This study was conducted in four villages: Mahlanya (26°29’S, 31°13’E), Sitjeni (26°28’S, 31°12’E), Mcaphozini (26°26’S, 31°12’E) and Elangeni (26°25’S, 31°13’E) at Lobamba, central Swaziland (690m above sea level). The area has been transformed into a matrix of small-scale subsistence farmland and farmers’ homesteads with none of the original natural vegetation remaining [33]. The staple crop is maize, with sorghum, vegetables and pulses grown on a smaller scale.

A typical homestead in this region supports 6–10 people [34]. Each homestead is made of a number of buildings clustered around a main house. Buildings range from thatched stick-and-mud to corrugated iron and tiled houses. The majority of homesteads engage in small-scale subsistence farming.

### Homestead survey

More than 200 homesteads from the four villages were visited as part of a survey on rodent management from which homesteads that keep dogs and/or cats were enumerated. We randomly selected ten homesteads with cats, ten with dogs, ten with both cats and dogs, and ten with neither cats nor dogs; these 40 homesteads formed the basis for this study. There was no difference in the breeds of cats (breed: mongrel Swazi) and dogs (breed: Africanis) used for the study and permission to carry out the research was granted by each head of household. Although we did not count the number of dogs and cats, at homesteads where they were present we generally saw between one and four dogs and one or two cats. These domestic dogs and cats were not chained up but were free to roam around the homestead and the surrounding fields.

A recent survey in the same area has convincingly shown that *Rattus rattus* was the dominant rodent in and around homesteads [33,35]. Nonetheless, using the same methodology we surveyed small mammals at these homesteads by setting Sherman live traps (HB Sherman Traps Inc., Tallahassee, Florida, USA) in areas of high activity over three consecutive nights in July 2015. Animals caught were identified and released. This survey was only conducted to assess whether the rodent community in these homesteads had recently changed. The ethics committee for the use of animals from the University of Swaziland approved the protocols used in this study which adhered to the guidelines of the American Society of Mammalogists for the use of wild mammals in research [36].

### Experimental design

**Fig 1:**
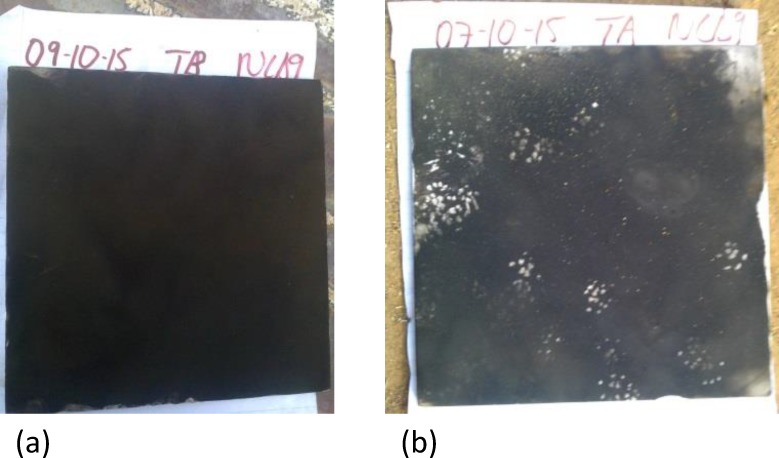
Rodent activity in homesteads was quantified by using tracking tiles. (a) blackened with soot; (b) marked with rodent footprints.

We quantified rodent activity at each homestead with the use of “tracking tiles”; white ceramic wall tiles (20cm x 20cm) that were blackened with soot using a smoking paraffin lamp (Fig 1). To measure the relative amount of rodent activity we calculated the percentage area of the tile covered by footprints [37]. We placed tracking tiles out for five consecutive nights in the cool dry season (July 2015) and again in the hot dry season (Oct 2015). We conducted live trapping based on the activity measures conducted in July. During both seasons we placed freshly sooted tiles out each evening, and removed the following morning. We determined the percentage area marked by rodent footprints by placing a transparent plastic sheet, divided into 16 squares (5cm x 5cm), on top of the tile. The number of squares with rodent footprints was expressed as a percentage of the total number of squares.

Employing feeding patches and measuring the GUD [14,38] we separately determined rodent foraging activity. We created feeding patches which consisted of plastic lunchboxes (size 7.1 cm x 23.0 cm x 17.4 cm) containing 1 kg of sand within which 50 peanuts were randomly buried. We placed feeding patches alongside the tracking tiles at each of the 40 homesteads for seven consecutive nights to allow rodents to acclimatise to them [39]. On the afternoon prior to the start of the experiment on day 8 we replenished the feeding patches. For five consecutive mornings we collected the feeding patches and counted the number of remaining peanuts and replaced missing ones.

### Analysis

We used a generalized-linear mixed model to examine differences in the levels of rat activity and giving-up densities at each homestead. We fitted both tracking tile activity and GUD to a negative binomial distribution using the glmmADMB package for R [40,41]. For tracking tile activity, we ran one model to examine the influence of treatment (none, cat, dog, both) and two additional models to determine if the influence of treatment varied by season (wet and dry). For the giving-up density models we only used homesteads that registered any feeding at the trays and included one variable as a fixed effect, i.e. treatment. For all models we included homestead as a random variable and set the treatment ‘none’ as the reference category. We considered treatments with beta estimates (β) and 95% Confidence Intervals that did not include 0 to be significantly different from the reference category. Using the SEpredicts command in the AICcmodavg package [42] we calculated the predicted estimates for activity and GUDs based on the best models. Additionally, to determine if the responses were influenced by their timing (i.e. day 1, 2, 3, 4 or 5) we used a likelihood ratio test to compare treatment models to a model that included the additional variable ‘day’, to account for the day the trial was conducted or the activity that was measured.

## Results

A total of 86 rodents of two species were captured within buildings and out-houses around homesteads in the study area. The majority (73) of specimens were *Rattus rattus*, with the remainder (13) being *Mastomys natalensis*.

**Fig 2:**
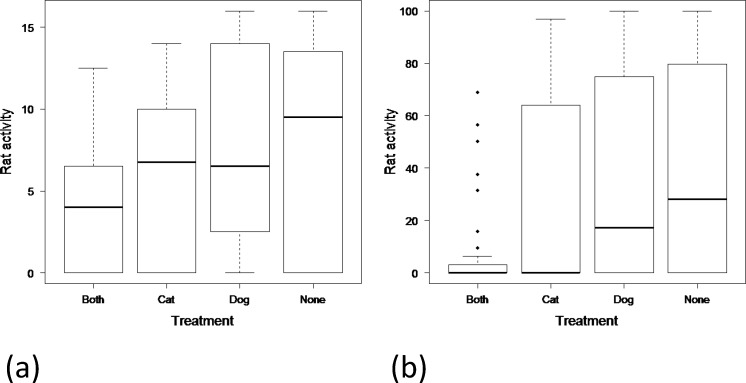
Boxplot showing median, and upper and lower quartiles, of rat activity around rural homesteads in Swaziland in the four treatments of this study averaged over the five nights. Treatments are with cats alone, with dogs alone, with both cats and dogs, and with neither cats nor dogs in: (a) July 2015; and (b) October 2015.

Rodent activity in homesteads was significantly reduced in the presence of both cats and dogs (β = -1.10 [-1.76- -0.43]) (S1 Table and Fig 2). Rodent activity in homesteads with cats alone (β = -0.27 [-0.94–0.40]) and dogs (β = -0.08 [-0.75- -0.59]) alone was also reduced, but not significantly so. The predicted number of tracking tiles marked by rodents in homesteads with both cats and dogs was 12 [5–26], with neither domestic predator present the predicted number of tracking tiles marked by rodents was 28 [18–43]. This pattern was consistent across seasons, with only the presence of both cats and dogs showing a significant reduction in rodent activity. The influence of cats and dogs appeared more pronounced in the wet season (β = -2.57 [-0.49- -4.65]) compared with the dry (β = -0.76 [-1.43- -0.09]). The timing (day) of activity measures did not improve the fit of the model (χ^2^ = 2.02, DF = 6, *p* = 0.156).

**Fig 3:**
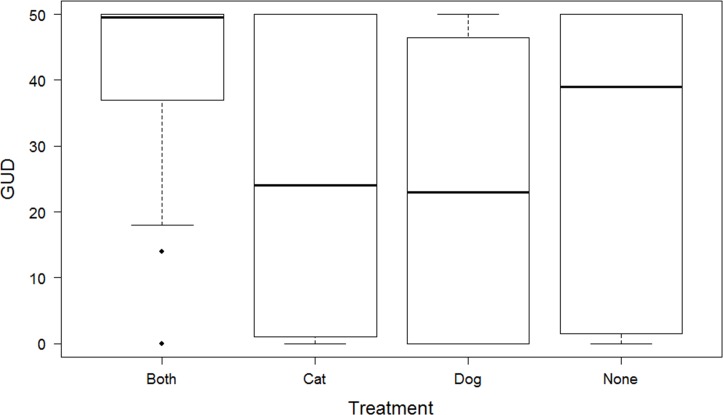
Boxplot showing median, and upper and lower quartiles, of rat giving up densities in rural homesteads in Swaziland in the four treatments of this study averaged over the five nights. Treatments were with cats alone, with dogs alone, with both cats and dogs, and with neither cats nor dogs.

Compared with no domestic predators in the homestead, the GUDs of rodents were significantly higher (fewer peanuts were eaten) at homesteads with both cats and dogs (β = -1.32 [-2.44- -0.20]) (Fig 3). The predicted giving-up densities of rodents at homesteads with neither cats not dogs was 30 peanuts [15–50], whereas it was 8 peanuts [2–32] at homesteads with both cats and dogs. There was no significant difference in GUDs of rodents at homesteads with cats alone (β = -0.11 [-1.13–0.91]) or dogs alone (β = -0.24 [-1.18- -0.70]) compared with GUDs of rodents from homesteads with neither cats nor dogs. Similar to the activity models, the timing (day) of GUDs measures did not improve the fit of the model (χ^2^ = 0.18, DF = 6, *p* = 0.671).

## Discussion

We showed that pest rodent activity was diminished in rural homesteads where both cats and dogs were present. Additionally, pest rodents foraged less where both these domestic predators were present. Thus, our study does not show dogs alone or cats alone to be important in affecting the landscape of fear of pest *Rattus rattus* and *Mastomys natalensis* in homesteads; however, we demonstrate there is a significant impact when the two predators are combined.

Previous studies have shown clear anti-predatory behaviours by rats and other taxonomic groups including Sykes’ monkeys, bottlenose dolphins, harbour seals, and dugongs [43,44,45]. Rats, in particular, respond to predators such as mongooses and particularly cats [2,5], the latter even eliciting an immunoreactivity response in the brain of the rat [6]. Dogs, on the other hand, do not seem to always elicit strong responses in rats, with some studies showing no effects on rodents exposed to dog faeces [46], whilst other studies have shown some consequences on rodent behaviour through the presence of dog integumentary odours [47]. The active removal of dogs from the environment has been shown to change rodent behaviour [48], although the mechanisms of this relationship are not understood and have not been investigated.

Contrary to our prediction, the greatest impact on pest rodents was the combined presence of cats and dogs. The behavioural response mechanisms that explain our results have yet to be determined. In addition to odour it is possible that rats could have been responding to visual cues. If visual cues were important, measures of cat and dog activity could potentially be used as an explanatory variable to explain variation in GUDs. Nonetheless, considerable evidence indicates that cats are important predators of rodents [22] and have exerted strong selective pressure on the behaviour and physiology of rats [6]. Evidence of the impact of dogs on rodents is more equivocal [25,49], and may be due to relatively agile and small-sized rats outmanoeuvring larger pursuit predators such as dogs. Alternatively, although we did not have different breeds of dogs in this study, dog breeds (e.g. compare Fox Terrier with Great Dane), may represent a greater variation in predator design (and perhaps behaviour) compared with cats which are built to more or less the same morphology. In any case, our data suggest that when a rat is confronted by both predators, each requiring perhaps a different anti-predator behavioural response, it creates a perceived environment where the risk of foraging is greater than the reward of acquiring the food resources; seen as reductions in activity and increases in GUDs. We suggest that there might be a synergistic influence of these two predators. In fact some sympatric predators such as wolves and coyotes [50] increase their territorial markings when they co-occur potentially increasing the landscape of fear for their prey.

Our study presents inferences based on correlational statistics. We recommend testing the impact of dogs and cats on pest rodent communities by conducting empirical trials to better understand the mechanisms involved. Furthermore, we did not measure the activity of the domestic dogs and cats in this study. We suggest that measuring such activity would only serve to confirm our conclusions.

## Supporting information

S1 Table Giving up densities of rodents at Lobamba, Swaziland. (CSV)
